# Social Support and User Characteristics in Online Diabetes Communities: An In-Depth Survey of a Large-Scale Chinese Population

**DOI:** 10.3390/ijerph17082806

**Published:** 2020-04-18

**Authors:** Dan Liang, Guanhua Fan

**Affiliations:** 1 MPH Education Center, Shantou University Medical College, 22 Xin Ling Road, Shantou 515041, China; 17dliang@stu.edu.cn; 2 Shantou University Medical College, 22 Xin Ling Road, Shantou 515041, China

**Keywords:** diabetes online community, online health communication behavior, health behavior, NCDs and health promotion, diabetes self management

## Abstract

*Objective*: To determine the characteristics of members of online diabetes communities as well as those factors affecting the provision and acceptance of social support. *Methods*: A cross-sectional STAR questionnaire survey was conducted among patients with diabetes who were members of online diabetes groups. Univariate and multivariate binary logistic regression analysis were adopted to explore the relative analysis of providing and accepting social support compared with the characteristics of members in virtual diabetics’ groups. *Results*: A total of 1297 respondents were collected. The map distribution of patients in China was mainly located in the Guangdong, Jiangsu, Shandong, Henan, and Hebei provinces. As for their demographic characteristics, respondents had diabetes or prediabetes and were between the ages of 21 and 50 years (Median age was 35.0 (interquartile range from 28.0 to 44.0)). Most respondents were married and lived in cities. The education level of patients was mainly distributed throughout junior high, technical secondary, high school, junior college, and undergraduate levels. Age, marital status, and education level varied by gender, and the total score of the patients aged 41 to 50 for social support had a statistical significance between male and female. In addition, when group members were in junior high school or below, or were undergraduate students, their total social support scores varied by gender. Binary logistic regression showed that in 21 independent variables the total score and the total score grade of relationship intensity in the online group and reorganize of age were significant. The patients’ social support acceptance of the map of respondents score grading of relationship intensity in the online group was 5.420 times higher than that of the lower score grading of relationship intensity in the group. At the same time, the patients’ social support acceptance of the patients at the age of less than or equal to 31 years old was 19.608 times higher than that of group members aged more than 31 years old. *Conclusion*: Age and education background of the patients affects scores of social supports between males and females. The higher the total score and the score grade of relationship intensity in the online group, the higher the patients’ social support acceptance. The younger patients had a better utilization of social support.

## 1. Introduction

With rapid developments in mobile Internet technology and the promotion and evaluation of the progress made by traditional mutual aid organizations, more people are participating in online peer-led social support groups. Such groups connect people in similar circumstances, particularly those grappling with a specific disease or personal challenge. This creation of online communities constitutes one important application of mobile Internet technology to solve problems and improve people’s quality of life [1,2]. The formation of online groups where people with diabetes can interact with each other has been a consequential development in diabetes management as it has been an “expansion and innovation” of the professional-led mode of intervention in traditional diabetes care. Considering these developments, we conducted a large-scale population survey of people with diabetes from China who were members of online diabetes communities (ODCs), we systematically collected data on the target population for empirical analysis, and the healthy social characteristics of the ODCs population were explored. This improved understanding of the relationship of personal and online interaction characteristics with acceptance of online social support in people with diabetes, thereby aiding diabetes treatment in general and the promoting the development of diabetes health groups in particular.

In mainland China, 623 million adults have diabetes or prediabetes: 130 million adults have diabetes, and 493 million adults in China (50.1% of the adult population) have prediabetes (i.e., impaired glucose tolerance) [3]. Furthermore, more people in China are getting diabetes, and the medical resources for diabetes treatment, primarily the supply of diabetes specialists, are gravely inadequate. Because of this inadequacy, patients are unlikely to receive effective and detailed patient education from professional medical staff during their regular clinical follow-up period. Thus, the people with diabetes are likely to have inadequate knowledge of diabetes, and daily blood glucose monitoring is likely to be discontinuous, non-compliant, and not standardized, thus introducing many high-risk potential threats in the daily self-management and prevention of complications among patients with diabetes [4,5].

Although online support organizations require users to own smartphones or computers, they transcend geographical limitations as well as those concerning social support infrastructures and the sizes of local communities. If Internet-based support organizations are found to be viable and effective, then their use should be advertised to increase the availability and diversity of communities that individuals can choose to join [6]. These practices would help to eliminate health disparities due to unequal access to health care resources and health information.

Diabetes is a chronic, incurable, and debilitating disorder; it is one of the most important risk factors for complications, and its consequences are irreversible [7]. In 2017, the International Diabetes Federation predicted that the number of people with diabetes in China aged 20–79 years will increase from 114.4 to 119.8 million by 2045. Additionally, the total medical expenditure on diabetes in 2017 was approximately 110 billion RMB, which was the second highest in the world [8]. In China, the economic burden of diabetes is only less than that of cancer and cardiovascular disease [9,10].

Diabetes treatment includes self-management methods, such as regular physical exercise, weight control, restricted caloric intake, and regular blood glucose monitoring. However, patient behavior necessitates change according to local conditions and changes in their daily routine and blood glucose level, which complicates the patient’s treatment plan [11,12]. As early as 1985, at least 1.5 million people used social support groups to cope with physical and psychological problems resulting from chronic diseases, including diabetes [13]. Social support has been demonstrated to have a positive and protective effect for patients with type 1 diabetes and high hemoglobin HbA1C levels, although such findings for patients with type 2 diabetes have been mixed [14,15,16]. Therefore, it is valuable to analyze the characteristics of providing and receiving social support and the characteristics of users in diabetes online communities.

## 2. Patients and Methods

### 2.1. Recruitment

In this study, we measured the characteristics of exchange of social support among patients with diabetes in online diabetes groups, with a focus on high-risk and critical groups. The online behavior scale for populations with diabetes [17,18,19,20,21,22,23] was adapted to a Chinese population of online-group users through a pretest. The overall alpha coefficient value of the data is 0.978, the alpha coefficient value of the social support part is 0.908, and the KMO value (Kaiser-Meyer-Olkin value) is 0.944. The contents of the scale are as follows: demographic data (gender, age, place of birth, education level, area of residence, marital status), course of disease, blood glucose level, disease effect, use of diabetes online health communities, motivation for group participation subscale (4 items; valid and reliable with Cronbach’s α = 0.872 > 0.800 and KMO = 0.826 > 0.800, respectively), self-management subscale (13 items; valid and reliable with Cronbach’s α = 0.938 > 0.900 and KMO = 0.966 > 0.800, respectively), online interaction subscale (8 items; valid and reliable with Cronbach’s α = 0.676 > 0.600 and KMO = 0.794 > 0.700, respectively), and online health community information dissemination factor subscale (15 items, including the strength of friendships made with other group members, information intensity, group professional intensity, recipient’s professional degree, and information collection; valid and reliable with Cronbach’s α = 0.916 > 0.900 and KMO = 0.958 > 0.900, respectively). After Bart’s sphere test, the cumulative variance interpretation rate value is 64.392%, which indicates that the data of this study are reliable and have a relatively excellent level of structural validity.

A cross-sectional survey using the STAR questionnaire was administered to patients with diabetes who were members of online groups over three months. Respondents were one of the following: (a) healthy young people living with older-adult relatives with diabetes, where these relatives either participated in online diabetes groups or shared diabetes-related information with family and friends through their smartphones; (b) patients with critical diabetes (fasting blood glucose level = 6.1–7.0 mmol/L, reduced glucose tolerance level > 7.8 mmol/L, or blood glucose level 2 h after a meal ≥11.1 mmol/L) [24] who had participated in online diabetes groups on QQ (TenCent Instant Message), WeChat, Chunyu Doctor, bulletin board systems, or Tieba; or (c) patients with diabetes who had participated in online diabetes groups. Because these three groups of people have experience in using online diabetes networks to interact with others and engage in peer education, they understand the relationship between providing and accepting social support as well as the characteristics of members in online diabetes groups.

Prospective respondents were included if they satisfied the following inclusion criteria: (a) they were patients with diabetes who had experience interacting with others in diabetes online communities; (b) they had experience interacting with people from high-risk groups or had low glucose tolerance (fasting blood glucose level = 5.6–6.1 mmol/L); or (c) they were young people with older-adult relatives with diabetes who needed help seeking counseling and maintaining their blood glucose levels.

Prospective respondents were excluded if they met any of the following exclusion criteria: (a) they accessed online diabetes communities through multiple (more than two) Internet protocol addresses; (b) their survey responses were contradictory; (c) they answered the questions randomly or had large sheets; (d) they answered “completely agree” for question 30, which indicated that the survey was completed by a machine and (e) they gave some variation of the following answer to the penultimate open-ended interview question, which is an open-ended discretionary response, written “Everyone should pay attention to diet control, engage in proper exercise, not stay up late, and prevent hypoglycemia and ketoacidosis”. After these exclusions, a total of 1297 valid questionnaires were collected.

We used the Likert scale to access social support scores and record basic information regarding eligible patients. The options were set to “fully agree”, “agree”, “uncertain”, “disagree” and “completely disagree” for “5 points”,”4 points”, “3 points”, “2 points” and “1 point”, separately in 3 dimensions (“Strength of the relationship in the online group (2 scoring items)”; “Information intensity (4 scoring items)”; and “Interaction with others in the online mobile environment (7 scoring items)”). We calculated the social support score of every eligible patient by 13 scoring items of this questionnaire. So, in this survey, the total score of each group member was 65 points and the lowest score was 13 points. If patients chose the options, “agree (5 points)” or “fully agree (4 points)” in every scoring item as a better score, that is to say, for the communication mode of diabetes online group, each research subject whose social support score was greater than 39 points was judged to have better acceptance and utilization of social support.

**Figure 1:**
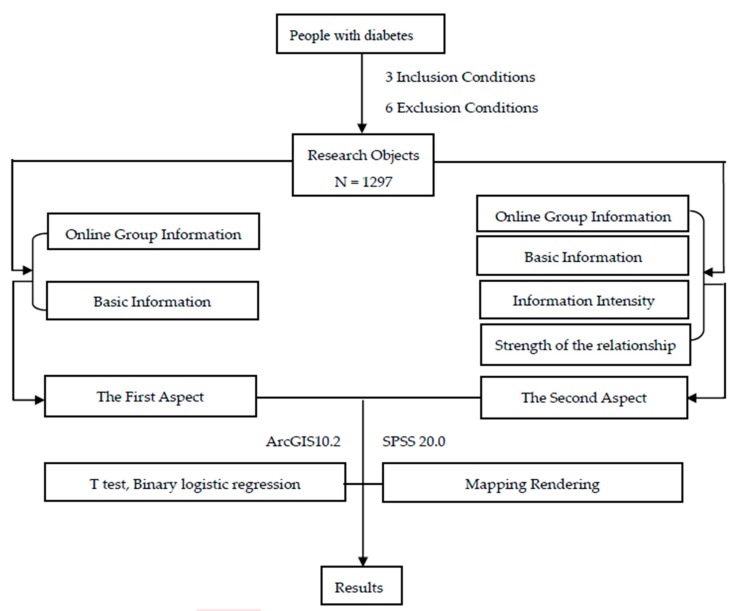
The flow chart of the entire research design.

The research design is illustrated in the following flow chart (Figure 1).

#### Ethics Approval and Consent to Participate

All subjects provided verbal informed consent to inclusion before they participated in the study, and the protocol was approved by the Ethics Committee of the Medical College of Shantou University (Code: SUMC-2016-39).

### 2.2. Research Design

The following data were analyzed.

(1) Demographic data concerning gender (X_1_), age (X_2_), marital status (X_3_), education level (X_4_), and area of residence (X_5_).
(2) Data regarding the following aspects of the diabetes online groups in which the respondent participated:
  (a) How much the respondent perceived the groups to be personally helpful for disease management (X_6_), rated “not helpful”, “slightly helpful”, “moderately helpful”, “very helpful”, or “extremely helpful”.
  (b) Average number of active members among the online diabetes groups in which the respondent had participated (X_7_): segmented into the seven ranges of <100, 101~, 301~, 501~, 1001~, 1501~, and 2001~.
  (c) The respondent’s membership status in the online diabetes groups (X_8_), categorized into the following:
    - long-term membership membership and active use of information but no basic interaction with other users;
    - active participation and communication, with great interest;
    - attempting to help others and vigorously exchanging ideas, whether adeptly or not;
    - long-term “diving” just to receive comfort and motivation from keeping up to date on happenings in the group;
    - keeping up to date with the direction of group interactions and content unrelated to one’s treatment plan;
    - long-term and active participation (communication and helping others) in the groups,
    - other statuses.
  (d) Data regarding whether the respondent had engaged in the following types of interaction in online diabetes groups (X_9_); the variables were binary (yes/no).
    - giving a thumbs up (X_9-1_),
    - uploading or viewing pictures (X_9-2_),
    - reading and using group information (X_9-3_),
    - viewing or replying to messages (X_9-4_),
    - sharing or viewing links (X_9-5_),
    - requesting help from online-group friends (X_9-6_),
    - exchanging feelings, experiences, or disease-management methods (X_9-7_), and communicating with online-group friends through private messaging (X_9-8_).
(3) The following was also assessed using a similar scale:
  (a) Strength of the relationship in the online group (X_10_):
    ① I haven’t met the group members who often share knowledge and experience with me. Although I don’t know group members directly, I trust them more (X_10-1_) and
    ② Friends with whom I often exchange knowledge and experience are people I have met (X_10-2_).
    Results were indicated as the following responses: fully agree, agree, uncertain, disagree, and completely disagree. The scores from “fully agree” to “completely disagree” are 5 to 1, at the same time, the total score of the two strengths of the relationship in the group was 12 points and it was classified into two grades; we defined the total score grades of X_10_ as X’_10_ in which “0” meant less than or equal to 6 points and “1” was for more than 6 points.
  (b) Information intensity (X_11_):
  (c)
    ① The exchange of knowledge and experience related to diabetes in the online group impressed me deeply (X_11-1_);
    ② The exchange of knowledge and experience related to diabetes in the group was persuasive to me (X_11-2_);
    ③ The group members who often exchange knowledge and experience have a serious and enthusiastic attitude (X_11-3_);
    ④ The group of friends who often exchange knowledge and experience sometimes communicate with me through private messages (X_11-4_).
    Results were indicated as the following responses: fully agree, agree, uncertain, disagree, and completely disagree. The scores from “fully agree” to “completely disagree” were 5 to 1, at the same time, the total score of the two strengths of the relationship in the online group was 20 points and it was classified into two grades; we defined the total scores grades of X_11_ as X’_11_ in which “0” meant less than or equal to 12 points and “1” was for more than 12 points.
(4) Data regarding the perceived depth of engagement with information from the online groups (Y, i.e., information intensity) were indicated by the extent of agreement with the following statements on the following scale:
  ① Ask relevant questions and answer questions about my medical condition to receive more responses for consulting information (Y_1_);
  ② Group members share my anxiety (such as unstable symptoms and poor treatment effects) (Y_2_);
  ③ Friends in the online group respect my life choices and decisions (Y_3_);
  ④ Group members make me feel valued (Y_4_);
  ⑤ I recognize the valuable information that group members communicate (Y_5_);
  ⑥ I can communicate more symptoms, conditions, or thoughts with group members (Y_6_);
  ⑦ When I am confused or uncomfortable, friends in the online group provide me with useful online assistance or information(Y_7_).
  Results were indicated as the following responses: fully agree, agree, uncertain, disagree, and completely disagree. The scores from “fully agree” to “completely disagree” are 5 to 1, at the same time, the total score of the seven interaction with others in the online mobile environment was 35 points and it was classified into two grades; we defined the total score grades from Y_1_ to Y_7_ as Y’ in which “0” meant less than or equal to 21 points and “1” was for more than 21 points.

### 2.3. Statistical Analysis

We analyzed the data to test two relationships. The scores differences and distributions of some independent variables from “X_1_” to “X_8_” between males and females; The independent variables from “X_1_” to “X_9_”, “X’_10_ “and “X’_11_” were included in the equation for binary logistic regression. The results from these two aspects were used to observe the relationships between providing and accepting social support and the characteristics of diabetic members in the group.

First, using a *t*-test or rank-sum test to calculate “Mean ± standard deviation” (X ± S) or “Median (Interquartile)” (M (Interquartile range (IQR): 25%–75%)) of every independent variable which depended on the distribution type of cases, number and scores of independent variables according to gender were collected. Then, binary logistic regression was used to get the OR value and its 95% CI to ascertain what factors affect the provision and acceptance of social support. Data were entered using Excel and analyzed using SPSS (Version 20.0). The map of the distribution of the regions in China where the participants came from was drawn using ArcGIS (Version 10.2). Two-tailed *p* values of <0.05 indicated statistical significance.

## 3. Results

### 3.1. Demographic Characteristics

Regarding demographic characteristics, 54% and 46% of respondents were male and female, respectively; 72.3% and 27.7% were married and single, respectively; and most respondents were between 21 and 50 years of age (The age distribution of the subjects did not conform to the normal distribution, because tests of normality (The test of using observation data to judge whether the population obeys normal distribution) showed the statistic for Shapiro-Wilk was 0.977 and *p* = 0.000), and median age was 35.0 (Interquartile ranged from 28.0 to 44.0), the age range in which diabetes is most likely to occur [25]. The education level accounting for the highest proportion of respondents was possession of an undergraduate degree (37.5%); 2.9% had a postgraduate degree and 26.8% had junior high school, technical secondary school, or high school qualifications, and 28.5% had junior college qualifications. Most (62.4%) lived in urban areas, whereas only 7.9% lived in villages. Results of the Chi-square test showed there was a statistical significance in age group (χ^2^ = 44.341, *p* = 0.000), martial status (χ^2^ = 23.879, *p* = 0.000) and education background level (χ^2^ = 15.845, *p* = 0.000) between men and women. No statistical significance was found in place of residence after participating in a diabetics’ group (such as QQ or WeChat group), the degree of help that improved their own diseases, the average number of members in various diabetics’ groups that participated with great attention and their own condition about diabetes (Table 1).

To observe whether there is a significant difference in social support scores of different sub groups between men and women under different demographic characteristics and joining group characteristics. Significant differences were observed in the scores of patients at the age of 41–50 (dispersion of social support scores in total patients: 35.00 (27.00, 51.00), dispersion of social support scores in total male patients: 28.00 (24.00, 31.00), dispersion of social support scores in total female patients: 51.50 (48.00, 53.00), and the *p* value was 0.000. No significant difference was observed in other independent variables between males and females (Table 2). The bracketed content in the Table 2 is the median of social support scores and IQR value of social support scores.

### 3.2. Binary Logistic Regression

#### 3.2.1. Univariate Binary Logistic Regression Analysis

First, we brought each independent variable from “X_1_” to “X_11_”, “X’_10_ “and “X’_11_” into the equation, i.e., binary logistic regression from which we preliminarily estimated factors related to independent variables (Y’) and the result is shown in Table 2. After that, all the dependent variables with statistical significance were included in the binary logistic regression to calculate odds ratio (OR) value, *p* value and 95% confidence interval (CI) of OR value and then correlation between dependent variables and independent variables was obtained by using the binary logistic regression analysis and the result was showed in Table 3. For X_2_, it had no 95%CI of Exp(B) (The exponent of B, OR) which indicated too few patients in each group, so X_2_ was subdivided into 2 groups, namely less than or equal to 31 years old and more than 31 years old and renamed X’_2_.

After each independent variable was brought into binary logistic regression equation, among these 21 independent variables, except for X’_2_, X_10_ (The total score in strength of the relationship in the group) and X’_10_ (The total score grade of X_10_), other factors were not statistically significant. There was a higher score for social support in the patients at the age of ≤ 31 years old, it was 14.925 times higher than that of patients older than 31 years old (Exp(B) = 0.067, 95%CI of Exp(B) = 0.041–0.110, *p* = 0.000). For “The total scores in strength of the relationship in the group”, the patients who chose “agree” or “fully agree” had more scores with options of “uncertain”, “disagree” and “completely disagree”, and the former was 2.672 times the latter (Exp(B) = 2.672, 95%CI of Exp(B) = 2.269–3.147, *p* = 0.000). Furthermore, Patients with “>6 points” were 9.100 times the group members with “≤6 points” in “X’_10_” (The total scores grade of X_10_) (Exp(B) = 9.100, 95%CI of Exp(B) = 5.906–14.023, *p* = 0.000) (Table 3).

#### 3.2.2. Multivariate Binary Logistic Regression Analysis

Finally, we put all independent variables with statistical significance, X’_2_, X_10_ and X’_10_ into the equation for the second time (Table 3). There was a higher score for social support in the patients at the age of ≤31 years old who were 19.608 times higher than patients older than 31 years old (Exp(B) = 0.051, 95%CI of Exp(B) = 0.027–0.097, *p* = 0.000). For “The total score in strength of the relationship in the group”, the patients who chose “agree” or “fully agree” had more scores than options of “uncertain”, “disagree” and “completely disagree”, and the former was 3.782 times the latter(Exp(B) = 3.782, 95%CI of Exp(B) = 2.849–5.020, *p* = 0.000). In addition, compared with the patients getting “>6 points (Exp(B) = 0.189, 95%CI of Exp(B) = 0.080–0.449, *p* = 0.000)” in “X’_10_ (The total scores grade of X_10_)”, the patients with “≤6 points” were more likely to obtain a higher scores (Table 4).

#### 3.2.3. Map of Respondents’ Region of Origin across China

**Figure 2:**
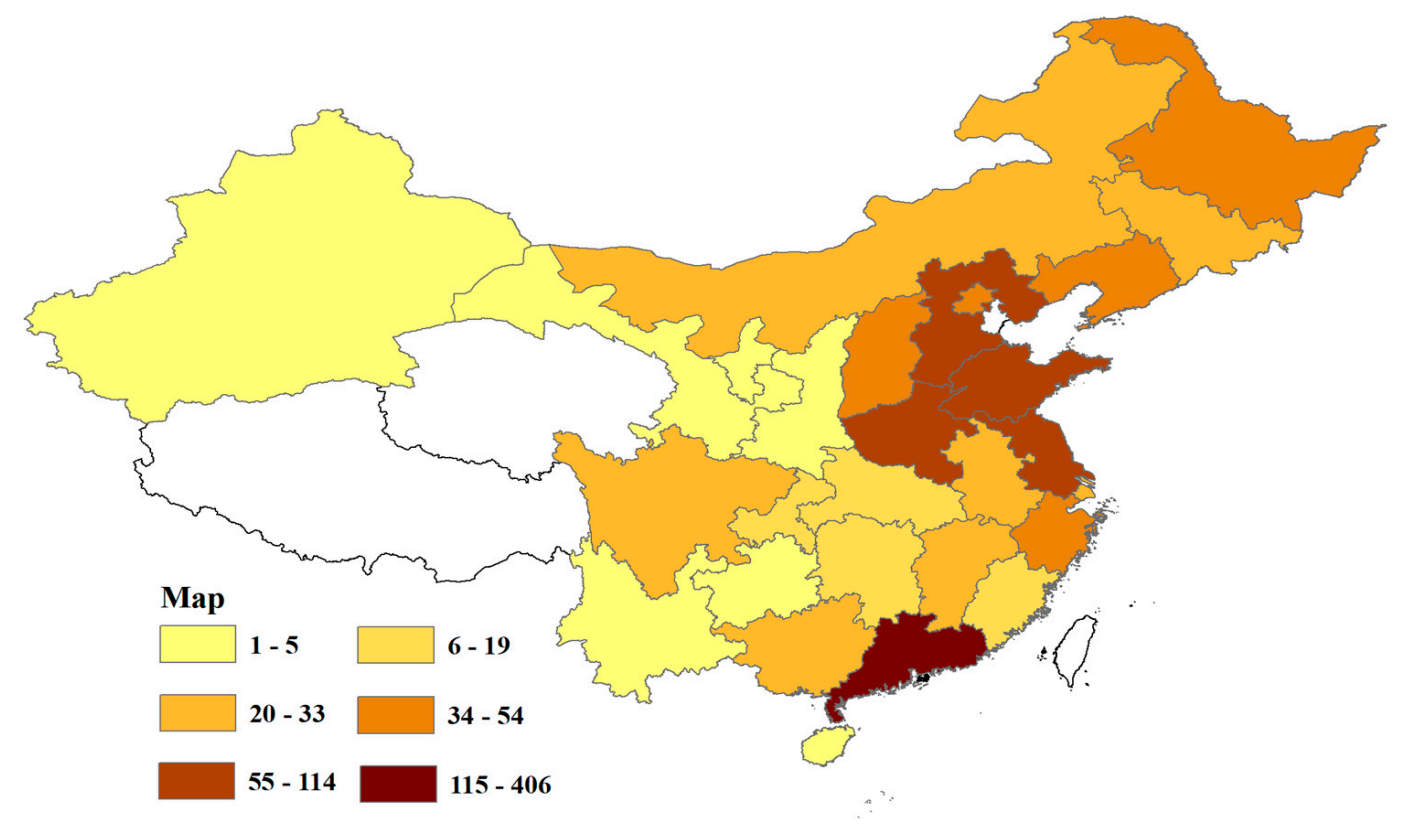
Map of the distribution of participants’ regions of origin across China.

We drew a map displaying the distribution of the regions in China from where the participants’ originated across China by using ArcGIS10.2 (Environmental Systems Research Institute, Redlands, California, USA), which more clearly shows the concentration area of patients and a comparative distribution of patients across the country (Figure 2). We can see the patient distribution from the map. Provinces where the respondents numbered 55 or more were Guangdong Province with 406 respondents, accounting for 31.59%; Jiangsu province with 114 respondents accounts for 8.87%; Shandong province with 99 respondents, accounts for 7.70%; Henan province (with 82 respondents accounts for 6.38%); and Hebei province (with 72 respondents accounts for 5.60%). In our survey, there was no participation from Taiwan, the Tibet autonomous region, the Macauo special administrative region, the Hong Kong special administrative region, Taiwan province, or Qinghai province (Figure 2).

## 4. Discussion

Social media has become a key medium through which people communicate and share their health experiences. Although opportunities for users to provide health-related content on personal website home pages have been available for a while [26], the diffusion and increasingly broad application of social media since the mid-2000s have made these opportunities much easier [27,28,29]. The creation of content through social media applications, such as WeChat, QQ, Sina microblog, Baidu Post Bar, and Facebook has become especially popular [30,31]. It has become a means to reflect their life and illness experience for numerous users. Simultaneously, this is also a way to build contact links among patients who can obtain information regarding related diseases and communicate with each other. Specifically, on social media, patients can share their personal doubts and their treatment experiences, including their experience regarding the efficacy of particular types of treatments. They can get social support, spiritual comfort, and guidance, which can serve to overcome depression, anxiety, or/and stress.

### 4.1. Basic Demographic Characteristics

In our study, there was no significant difference in the composition of patients between men and women, but studies have shown that although our study had an almost equal number of men and women, the burden of traditional risk factors for diabetes has been demonstrated to be greater in women than men. Prospective studies have demonstrated that, relative to men, high blood pressure, low HDL (High-density lipoprotein) cholesterol, and high triglyceride levels in women are more strongly associated with an increased risk of diabetes-related coronary heart disease (CHD) in women. However, after adjustment for traditional risk factors, a substantial proportion of diabetes-related CHD risk remains unexplained in both sexes [32], indicating that although women have a higher risk of complications after developing diabetes, this risk does not differ between men and women.

Regarding age composition, respondents were mainly concentrated in the age group of 21–50 years old. However, although age and diabetes type are closely related, respondents were not separated according to specific classifications of diabetes type in this study. Although children are being diagnosed at progressively younger ages [33], no children younger than 11 years were included. Children under 11 were excluded because they were not considered active social media users and the likelihood of developing diabetes increases as they age as a result of poor eating habits (such as a high sugar and fat diet), lack of exercise, as well as intervening viral and genetic factors [34] (WHO, 2006). Furthermore, the proportion of patients aged 71 years or older was only 1% of respondents. This was likely because various complications related to aging or diabetes tend to prevent people with diabetes from living beyond the age of 71, making this age group the lowest percentage [35,36,37,38].

By Chi-square test, statistical difference was shown in age, martial status and education background level between men and women in our study. Age is an important factor for diabetes mellitus and age distribution of diabetes in China increases with age. Nevertheless, there is a trend to diabetes developing at younger ages. The number of patients aged 21 to 40 distinctly increased in Table 1, and the reasons may be related to the following factors: a. young and middle-aged people undertake a great responsibility in society and with their family, as well, they suffer an economic burden and a household burden in supporting their children and parents; b. young and middle aged people are the mainstays of society, as such, they work under pressures that can create negative mental states and emotions; c. in addition, they have bad habits, such as smoking, drinking and eating a high-sugar, high-fat diet leading to metabolic disorders.

At the same time, this study found the social support scores of males higher than those of females for the ages of 41 to 50 and for 51 to 60. This may be a result of men and women having different social roles in China. Housework is mainly undertaken by women which causes them to have less time time than men to discuss their condition and experience of diabetes with other patients. In terms of educational background, the scores for social support of males younger than high school age and during the undergraduate years were higher than those of females. This may be because, unlike females, during these periods males are more reluctant to establish face-to-face contact with peers and may feel more comfortable with online discussion [39,40].

### 4.2. Binary Logistic Regression

After univariate and multivariate binary logistic regression analysis, we found some factors related to scores for social support and obtained the corresponding OR value and its 95% CI.

First, there was a higher score for social support in patients aged ≤31 years of 14.925 and 19.608 times higher than patients older than 31 years in univariate and multivariate binary logistic regression analysis, respectively. Although there is a trend towards younger diabetic patients, type 2 diabetes is more common in the elderly. Diabetes puts them under sustained psychological pressure creating a lack of confidence and enthusiasm for life, reduces the social activities in which they participate and increases both a reluctance to disturb others and seek out social support resources. It is the younger people who have more opportunities and energy to access social intercourse apps, as well, and they find it easier to find additional channels of communication than older people regarding their health status. It may be for this reason that patients older than 31 years received more social support and had a higher utilization of social support. Secondly, the higher the total score and strength of the relationship with the online group the higher the social support score of group members. The strength of relationships indicated the level of basic trust among group members in the online group, shown in the intensity of chatting with one another. Those group members who spent more time sharing with companions regarding self-care, medication, treatment experiences, health status, developing status of their diabetes, or merely venting their frustrations, gained and provided more experiences of assistance with respect to group members. In the online atmosphere, patients were able to release negative emotions and find solutions and comfort. Furthermore, by developing strong relationships, patients were able to supervise each other to complete tasks and learn better lifestyle treatment options. Finally, it should be noted that most participants were from the Guangdong and Jiangsu provinces—regions with more developed economies—likely resulting in residents having poorer eating habits from a richer diet, insufficient time for exercise, as well as a busier lifestyle.

## 5. Limitations

However, this study presented some limitations: firstly, online surveys are easily ignored by people with diabetes, which makes the recovery of questionnaires more difficult. We recommend that online questionnaires be conducted in conjunction with the moderators of online health communities, especially with regard to their distribution, which would make it easier to motivate more people with diabetes to complete the questionnaires. In addition, to increase the reliability and rigor of the research results and reduce the influence of irrelevant variables (such as whether the respondents had diabetes and how long they had been members of the group), this study had defined inclusion criteria for participation, The respondents should have enough experience in participating in the online diabetes community, and thus the people with diabetes but without online community experience were not included.

Secondly, people with diabetes who are less educated, older or younger, and in rural areas have very limited access to online social support, which may lead to selection bias in the study.

Finally, the questionnaire is self-evaluating for online social support behavior and the degree of improvement of one’s own disease. Therefore, if long-term follow-up of the people with diabetes can be carried out, the accuracy of the results will definitely be improved, and at the same time it will be helpful to the improvement in the quality of life of the Chinese diabetes population.

## 6. Conclusions

The nature of diabetes means that diabetic patients need long-term daily management of their disease. Participants in online groups can help with this type of management. The purpose of this survey was to investigate the characteristics of members of online diabetes groups and their relationship to providing or accepting social support in online diabetes groups. It was found that the higher the total score and the strength of the total score grade regarding the relationship to the online group determined the level of social support group members gained and the utilization of social support. Medical staff, general practitioners and public health workers need to have ways to help the diabetic population manage their care more skillfully. Online mutual aid platforms have been shown to be an effective method for promoting this more skillful management.

## Figures and Tables

**Table 1:** Chi-square result of basic information between male and female.

Characteristics	ALL (N = 1297) n (%)	Male (N = 700) n (%)	Female (N = 597) n (%)	χ^2^	Sig.
**Age group**				44.341	0.000 *
11–20	76 (5.9)	56 (8.0)	20 (3.4)		
21–30	349 (26.9)	206 (29.4)	143 (24.0)		
31–40	459 (35.4)	203 (29.0)	256 (42.9)		
41–50	256 (19.7)	134 (19.1)	122 (20.4)		
51–60	119 (9.2)	82 (11.7)	37 (6.2)		
61–70	25 (1.9)	13 (1.9)	12 (2.0)		
≥71	13 (1.0)	6 (9)	7 (1.2)		
**Martial status**				23.879	0.000 *
Single	359 (27.7)	233 (33.3)	126 (21.1)		
Married	938 (72.3)	467 (66.7)	471 (78.9)		
**Education background level**				15.845	0.003 *
Junior high school or below,	56 (4.3)	29 (4.1)	27 (4.5)		
Technical secondary school, Or High school	348 (26.8)	197 (28.1)	151 (25.3)		
Junior college	369 (28.5)	168 (24.0)	201 (33.7)		
Undergraduate	487 (37.5)	285 (40.7)	202 (33.8)		
Master degree or above	37 (2.9)	21 (3.0)	16 (2.7)		
**Place of Residence**				1.271	0.530
City	809 (62.4)	428 (61.1)	381 (63.8)		
Town	385 (29.7)	217 (31.0)	168 (28.1)		
Countryside	103 (7.9)	55 (7.9)	48 (8.0)		
**Improvement**				2.908	0.573
Little help	55 (4.2)	34 (4.9)	21 (3.5)		
A little help	368 (28.4)	200 (28.6)	168 (28.1)		
Help to a certain extent	429 (33.1)	223 (31.9)	206 (34.5)		
Help larger	281 (21.7)	149 (21.3)	132 (22.1)		
Help a lot	164 (12.6)	94 (13.4)	70 (11.7)		
**Number of Group members**				9.864	0.130
≤100	344 (26.5)	166 (23.7)	178 (29.8)		
101–300	399 (30.8)	227 (32.4)	172 (28.8)		
301–500	334 (25.8)	183 (26.1)	151 (25.3)		
501–1000	120 (9.3)	62 (8.9)	58 (9.7)		
1001–1500	46 (3.5)	26 (3.7)	20 (3.4)		
1501–2000	45 (3.5)	30 (4.3)	15 (2.5)		
≥2001	9 (0.7)	6 (0.9)	3 (0.5)		
**Health status**				5.775	0.329
Diabetes, live Normally	437 (33.7)	222 (31.7)	215 (36.0)		
Prediabetes, live Normally	519 (40.0)	290 (41.4)	229 (38.4)		
High risk diabetes, live Normally	205 (15.8)	119 (17.0)	86 (14.4)		
Diabetes, need temporary rest	51 (3.9)	27 (3.9)	24 (4.0)		
Diabetes, need permanent rest	29 (2.2)	12 (1.7)	17 (2.8)		
No diabetes	56 (4.3)	30 (4.3)	26 (4.4)		

Note: “*” means a statistically significant result.

**Table 2:** The distribution of basic information and social support scores in every case between male and female.

Characteristics	Total (Median, (IQR))	Male (Median_1_, (IQR_1_))	Female (Median_2_, (IQR_2_))	U	Sig.
**Age Group**					
11–20	76	56	20	514.50	0.589
	(50.50 (48.00, 53.75))	(50.00 (48.00, 53.75))	(52.00 (49.00, 53.75))		
21–30	349	206	143	13,821.00	0.324
	(52.00 (49.00, 53.00))	(52.00 (49.00, 54.00))	(52.00 (49.00, 53.00))		
31–40	459	203	256	25,849.00	0.923
	(52.00 (49.00, 54.00))	(52.00 (49.00, 54.00))	(52.00 (49.00, 54.00))		
41–50	256	134	122	0.000	0.000 *
	(35.00 (27.00, 51.00))	(28.00 (24.00, 31.00))	(51.50 (48.00, 53.00))		
51–60	119	82	37	1510.00	0.968
	(52.00 (49.00, 54.00))	(52.00 (49.00, 54.00))	(52.00 (49.00, 54.00))		
61–70	25	13	12	71.50	0.721
	(52.00 (49.00, 53.00))	(52.00 (49.00, 53.00))	(51.50 (48.25, 53.75))		
≥71	13	6	7	13.50	0.277
	(53.00 (51.50, 54.00))	(52.50 (47.75, 54.00))	(53.00 (52.00, 55.00))		
**Martial Status**					
Single	359	233	126	13,470.00	0.195
	(52.00 (49.00, 54.00))	(52.00 (49.00, 54.00))	(52.00 (48.75, 53.00))		
Married	938	467	471	108,499.00	0.720
	(52.00 (49.00, 54.00))	(52.00 (49.00, 54.00))	(52.00 (49.00, 54.00))		
**Education Background**					
Junior high school or below	56	29	27	299.50	0.128
	(52.00 (49.00, 53.00))	(52.00 (49.00, 54.00))	(51.00 (48.00, 52.00))		
Technical secondary school, or High school	348	197	151	14,417.00	0.620
	(52.00 (49.00, 54.00))	(52.00 (49.00, 54.00))	(53.00 (49.00, 54.00))		
Junior college	369	168	201	16,333.50	0.587
	(52.00 (49.00, 53.00))	(52.00 (49.00, 53.00))	(52.00 (49.00, 53.00))		
Undergraduate	487	285	202	28,023.00	0.616
	(52.00 (49.00, 53.00))	(52.00 (49.00, 53.00))	(52.00 (49.00, 54.00))		
Master degree or above	37	21	16	152.00	0.621
	(51.00 (49.00, 54.00))	(51.00 (49.00, 54.00))	(51.00 (49.00, 53.00))		
**Place of Residence**					
City	809	428	381	80,902.50	0.848
	(52.00 (49.00, 53.50))	(52.00 (49.00, 54.00))	(52.00 (49.00, 53.00))		
Town	385	217	168	18,161.00	0.950
	(52.00 (49.00, 54.00))	(52.00 (49.00, 54.00))	(52.00 (49.00, 54.00))		
Countryside	103	55	48	1235.50	0.573
	(52.00 (49.00, 54.00))	(52.00 (49.00, 54.00))	(52.00 (49.00, 54.00))		
**Improvement**					
Almost no help	55	34	21	355.50	0.979
	(52.00 (49.00, 53.00))	(52.00 (49.00, 53.25))	(52.00 (49.00, 53.50))		
A little help	368	200	168	15,481.50	0.195
	(52.00 (48.00, 54.00))	(52.00 (48.00, 54.00))	(52.00 (48.00, 53.75))		
Help to a certain extent	429	223	206	21,912.00	0.407
	(52.00 (49.00, 53.00))	(52.00 (49.00, 53.00))	(52.00 (49.00, 53.00))		
Help a significat extent	281	149	132	9786.50	0.944
	(52.00 (49.00, 54.00))	(52.00 (49.00, 54.00))	(52.00 (49.00, 54.00))		
Help a lot	164	94	70	3268.50	0.943
	(52.00 (49.00, 54.00))	(52.00 (49.00, 54.00))	(52.00 (49.00, 54.00))		
**Number of Group Members**					
≤100	344	166	178	13,839.50	0.307
	(52.00 (49.00, 54.00))	(52.00 (49.00, 54.00))	(52.00 (48.00, 53.00))		
101–300	399	227	172	13,747.00	0.097
	(51.00 (48.75, 53.00))	(51.00 (48.00, 53.00))	(51.50 (49.00, 53.00))		
301–500	333	183	150	13,609.00	0.894
	(52.00 (49.00, 54.00))	(52.00 (49.00, 54.00))	(52.00 (49.00, 54.00))		
501–1000	121	62	59	1482.00	0.070
	(52.00 (49.00, 54.00))	(51.00 (48.75, 53.00))	(53.00 (50.00, 54.00))		
1001–1500	46	26	20	207.00	0.238
	(51.00 (49.00, 53.25))	(51.00 (49.00, 53.00))	(52.00 (49.25, 54.75))		
1501–2000	45	30	15	216.50	0.837
	(52.00 (49.50, 53.00))	(52.00 (49.00, 53.00))	(51.00 (50.00, 54.00))		
≥2001	9	6	3	5.00	0.291
	(53.00 (50.50, 54.00))	(53.50 (52.00, 54.25))	(52.00 (44.00, 54.00))		
**State in the Group**					
Long term attention but little or no communication	266	140	126	8684.50	0.827
	(52.00 (49.00, 54.00))	(52.00 (49.00, 54.00))	(52.00 (49.00, 54.00))		
If having interesting experience, actively participate in the exchange	570	288	282	38,970.00	0.402
	(52.00 (49.00, 53.00))	(52.00 (49.00, 54.00))	(52.00 (49.00, 53.00))		
Strive to help others and actively exchange ideas whether good at it or not	317	189	128	11,273.00	0.301
	(52.00 (49.00, 54.00))	(52.00 (49.00, 53.00))	(52.00 (49.00, 54.00))		
Long term “Silence”, only get comfort and motivation from the group direction	94	55	39	901.00	0.185
	(52.00 (49.00, 53.00))	(52.00 (49.00, 54.00))	(52.00 (49.00, 53.00))		
If time is available, view group direction interaction unrelated to their treatment plan	43	23	20	197.50	0.425
	(52.00 (49.00, 54.00))	(52.00 (49.00, 53.00))	(52.00 (49.00, 54.00))		
Long term attention, active communication and help others	1	1	0	-	-
	(54.00 (54.00, 54.00))	(54.00 (54.00, 54.00))	-		
Other	6	5	1	0.000	0.137
	(53.00 (47.50, 55.00))	(54.00 (50.50, 55.00))	(43.00 (43.00, 43.00))		

Note: IQR: inter-quartile range, the disparity between Q3 and Q1 (Q1 = the 25th percentile of all values in the sample after ranking from small to large; Q3 = the 75th percentile of all values in the sample after ranking from small to large). In this study, it refers to the IQR of social support scores for the overall subjects. IQR_1_: it is equal to the IQR of social support scores for male subjects. IQR_2_: it is equal to the IQR of social support scores for female subjects. Median: the number in the middle of a set of data in order. It means the Median of social support scores for the overall subjects in this research. Median_1_: it means the Median of social support scores for male subjects in this research. Median_2_: it means the Median of social support scores for female subjects in this research. U: U test (Z test), it is a nonparametric test used to evaluate whether two independent samples come from the same population.

**Table 3:** The results of univariate binary logistic regression analysis for social support in patients.

Independent Variable	B	Exp(B)	95% CI of Exp(B)	Sig.
**Gender**				
Male		1.000		-
Female	−0.314	0.730	0.508–1.050	0.090
**Age Group**				
11–20		1.000		-
21–30	−19.196	0.000	-	0.999
31–40	−18.802	0.000	-	0.999
41–50	−19.008	0.000	-	0.999
51–60	−19.425	0.000	-	0.999
61–70	−18.573	0.000	-	0.999
≥71	−18.025	0.000	-	0.999
**Regrouping of Age**				
≤31 years old		1.000		-
>31 years old	−2.700	0.067	0.041–0.110	0.000 *
**Marital Status**				
single		1.000		-
married	−0.042	0.959	0.637–1.442	0.839
**Education Background Level**				
Junior high school or below		1.000		-
Technical secondary school, or High school	0.478	1.613	0.478–5.447	0.441
Junior college	0.917	2.501	0.953–6.559	0.062
Undergraduate	0.143	1.154	0.459–2.899	0.761
Master degree or above	0.771	2.163	0.852–5.493	0.105
**Place of Residence**				
city		1.000		-
town	0.738	2.091	0.826–5.292	0.119
village	−0.265	0.767	0.523–1.127	0.177
**After Participating in a Diabetics’ Group, the Degree of Help that Improved Their Own Diseases**				
almost no help		1.000		-
a little help	0.628	1.874	0.525–6.692	0.334
a certain degree of help	0.028	1.029	0.552–1.917	0.929
a significant degree of help	−0.222	0.801	0.443–1.450	0.464
a lot of help	0.102	1.107	0.573–2.140	0.762
**The Average Number of Members in Various Diabetics’ Groups that Participated**				
≤100		1.000		-
101–300	−0.051	0.950	0.116–7.795	0.962
301–500	0.034	1.035	0.126–8.475	0.975
501–1000	0.095	1.099	0.133–9.057	0.930
1001–1500	0.442	1.556	0.175–13.857	0.692
1501–2000	1.012	2.750	0.222–34.038	0.431
≥2001	1.705	5.500	0.311–97.232	0.245
**The Status after Joining an Online Diabetics’ Group**				
Long-term membership and attention and actively access information and knowledge but little or no basic exchange		1.000		-
If interested and having a good experience in the group, actively participating and communicating	−0.050	0.951	0.798–1.133	0.573
**Interaction within an Online Diabetics’ Group**				
**Thumbs up**				
yes		1.000		-
no	−0.135	0.874	0.5240–1.457	0.605
**Upload or view pictures**				
yes		1.000		-
no	−0.073	0.930	0.582–1.485	0.761
**Read and use group information**				
yes		1.000		-
no	0.011	1.011	0.563–1.814	0.972
**Reply or view messages**				
yes		1.000		-
no	0.060	1.062	0.607–1.858	0.833
**Exchange or view links**				
yes		1.000		-
no	−0.253	0.776	0.479–1.260	0.306
**Request group friends to provide help**				
yes		1.000		-
no	0.474	1.606	0.885–2.916	0.120
**Exchange feelings, experiences, or methods**				
yes		1.000		-
no	−0.002	0.998	0.540–1.846	0.996
**Communicate with group friends through private messages**				
yes		1.000		-
no	0.218	1.243	0.792–1.953	0.344
**The total score in Strength of the relationship in the group**	0.983	2.672	2.269–3.147	0.000 *
**The total score grade of the total score in strength of the relationship in the group**				
≤6 points		1.000		-
>6 points	2.208	9.100	5.906–14.023	0.000 *
**The total scores in Information intensity**				
The exchange of knowledge and experience related to diabetes in the group impressed me deeply		1.000		-
The exchange of knowledge and experience related to diabetes in the online group was persuasive to me	−18.376	0.000	0.000	0.961
**The total score grade of the total scores in information intensity**				
≤12 points		1.000		-
>12 points	−19.111	0.000	0.000	0.996

Note: “*” means a statistically significant result. B: Partial regression coefficient. Exp(B): The exponent of B, it refers to “OR” value.

**Table 4:** The results of multivariate binary logistic regression analysis for social support in patients.

Independent Variable	B	Exp(B)	95%CI of Exp(B)	Sig.
**Regrouping of Age**				
≤31-year-old		1.000		-
>31-year-old	−2.972	0.051	0.027–0.097	0.000 *
**The Total Score in Strength of the Relationship in the Group**				
I haven’t met the group members who often share knowledge and experience with me		1.000		-
Friends with whom I often exchange knowledge and experience are people I have met	1.330	3.782	2.849–5.020	0.000 *
**The Total Scores Grade of the Total Score in Strength of the Relationship in the Group**				
≤3 points		1.000		-
>3 points	1.690	5.420	2.320–12.659	0.000 *

**Note**: “*” means a statistically significant result.

## Data Availability

The datasets used and analyzed during the current study are available from the corresponding author on reasonable request.
